# Supplementary material for: Quantification of Signal Transduction Pathway Activity in Pancreatic Ductal Adenocarcinoma
Source: Int J Mol Sci. 2025 Nov 25;26(23):11385. doi: 10.3390/ijms262311385 (PMC12692056; doi:10.3390/ijms262311385)
Supplement: Supplementary file 1 [file ijms-26-11385-s001.zip › ijms-3936354-supplementary.pdf]

Supplemental Figures

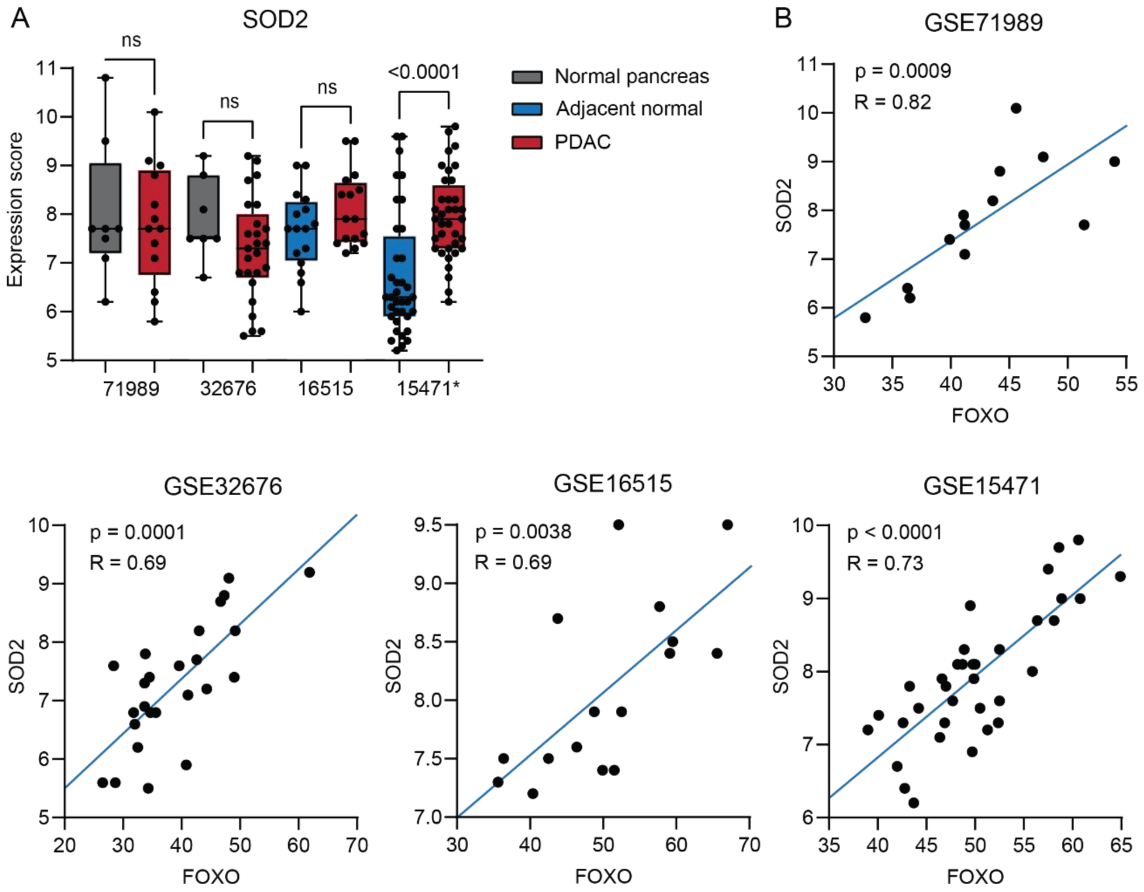

Supplemental Figure S1. (**A**) SOD2 expression of pancreatic ductal adenocarcinoma (PDAC) compared with normal pancreas (unpaired, GSE71989 and GSE32676) and normal adjacent pancreas (paired, GSE16515 and GSE15471). p value > 0.01 is considered non-significant (ns). p values ≤ 0.05 are depicted in numbers. (**B**) Correlation FOXO STP activity to oxidative stress, measured by mRNA expression level of superoxide dismutase 2 (SOD2).

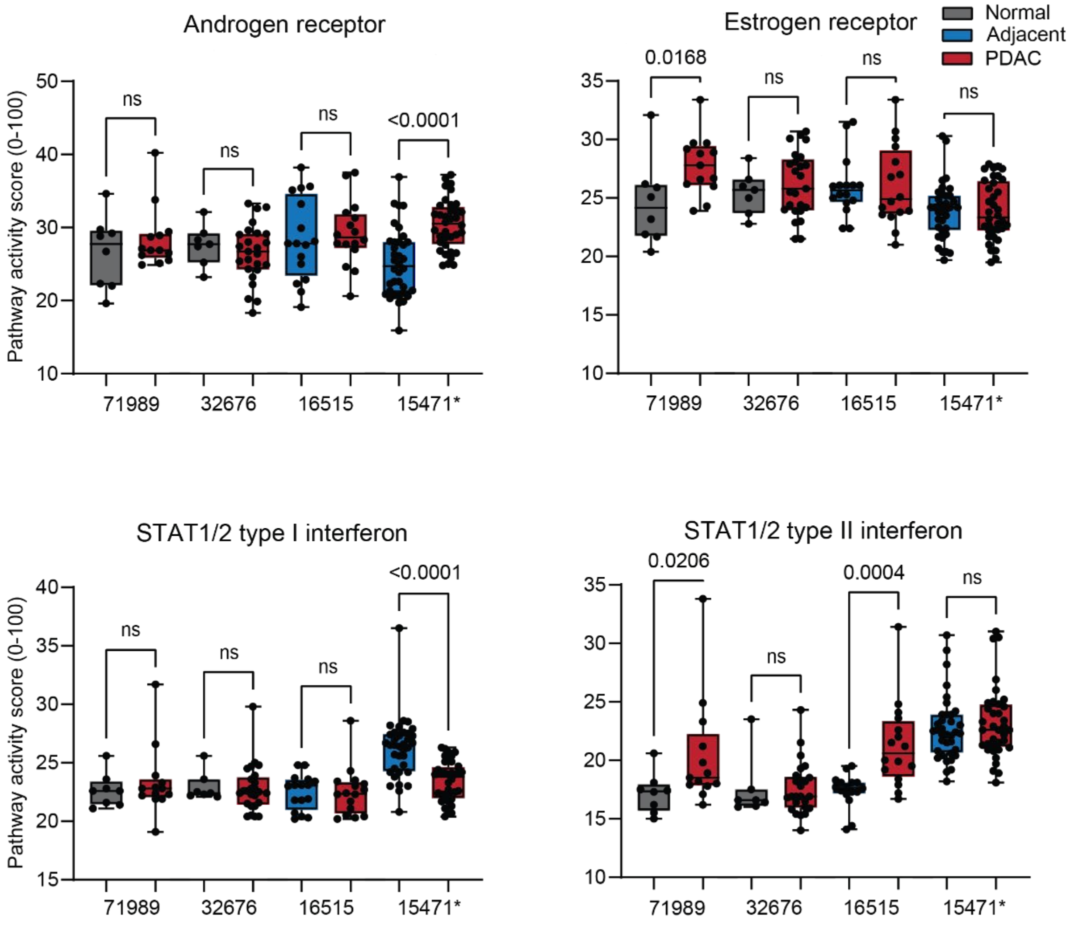

Supplemental Figure 2. Signal transduction pathway (STP) activity of the androgen receptor (AR), estrogen receptor (ER), JAK-STAT1/2 type I and JAK-STAT1/2 type II interferon STPs in pancreatic ductal adenocarcinoma (PDAC). PDAC is compared with normal pancreas (unpaired, GSE71989 and GSE32676) and normal adjacent pancreas (paired, GSE16515 and GSE15471). $p > 0.01$ is considered non-significant (ns). p values are depicted in numbers if $p \leq 0.05$.

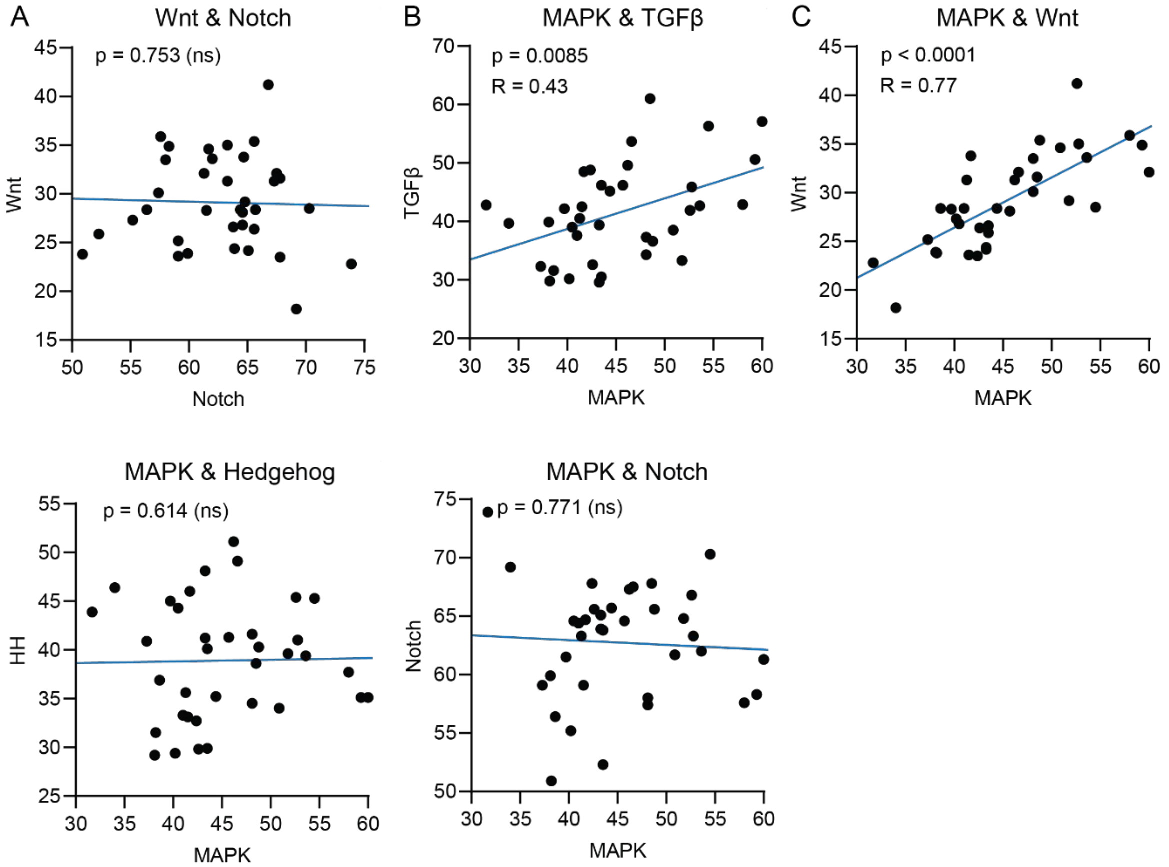

Supplemental Figure 3. Correlation of STP activity scores from PDAC tumor samples from GSE15471. (**A**) Correlation of Wnt signal transduction pathway (STP) activity with Notch. (**B**) Correlation of MAPK STP activity in tumor samples with TGFβ and (**C**) Wnt, Notch and Hedgehog (HH).

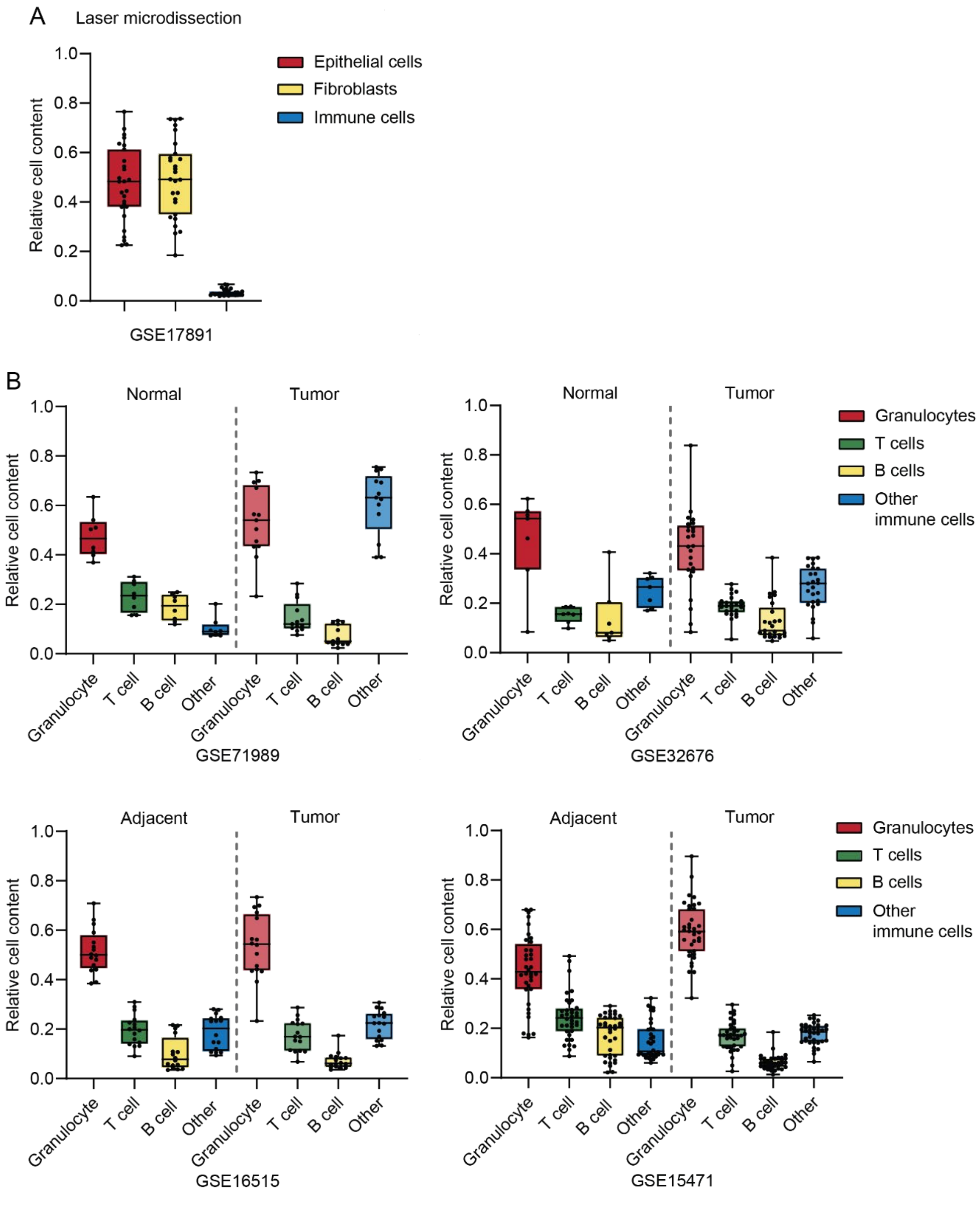

Supplemental Figure 4. Relative cell type content of samples using deconvolution software. (**A**) Relative cell type content of laser microdissected PDAC tumor samples from GSE17891. (**B**) Analyses of the immune cell content (total = 1.0) to determine which subsets of immune cells are present in the samples.

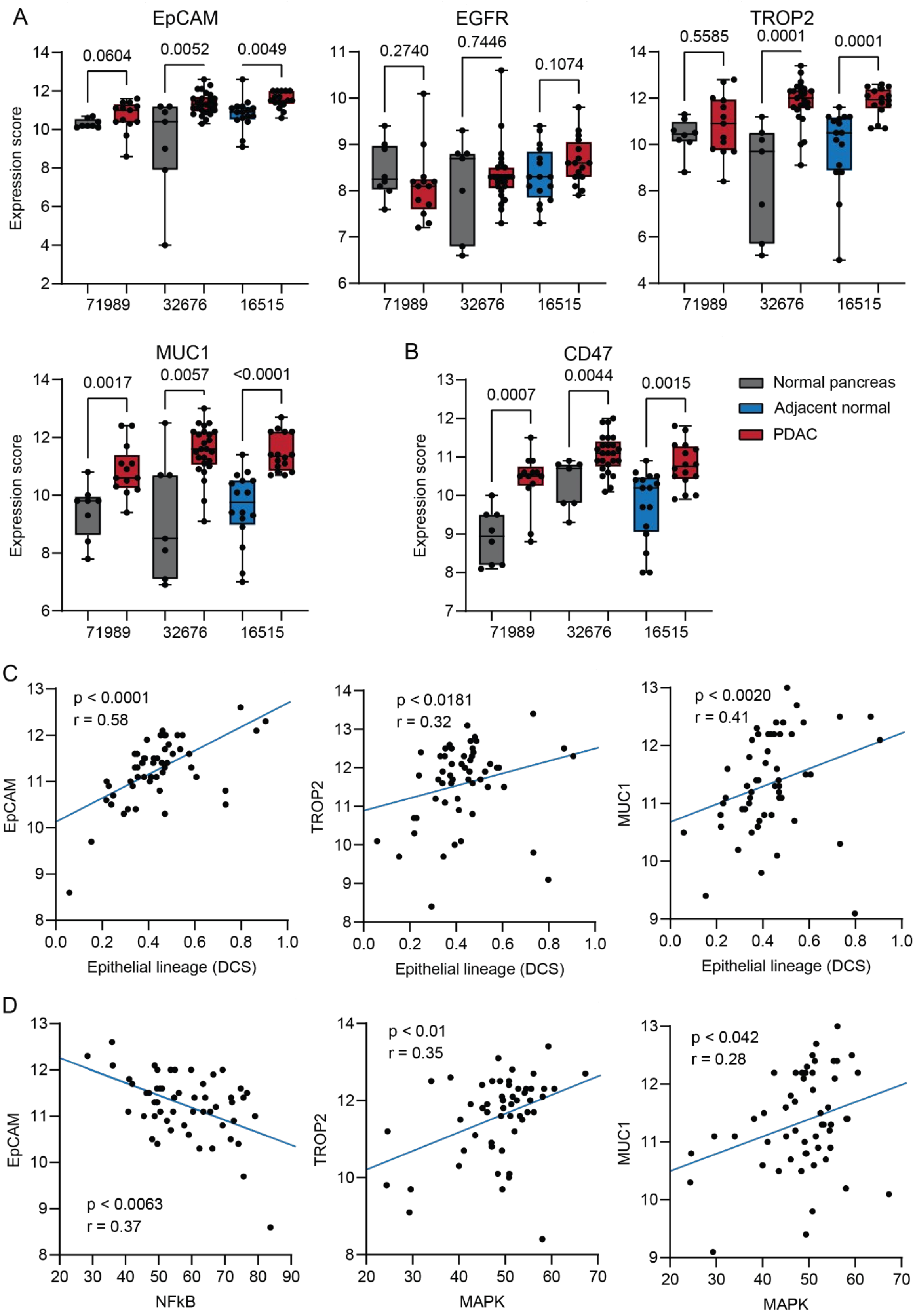

Supplemental Figure 5. (**A**) Gene expression of the tumor-associated antigens (TAAs) EpCAM, EGFR, TROP2 and MUC1 comparing normal and tumor-adjacent normal pancreas with PDAC. (**B**) Gene expression of CD47 comparing normal or adjacent tissue with PDAC. (**C**) Correlation of TAAs higher expressed in PDAC with relative epithelial cell content as determined with deconvolution software (DCS). (**D**) Significant correlations of TAAs with signal transduction pathways activity.

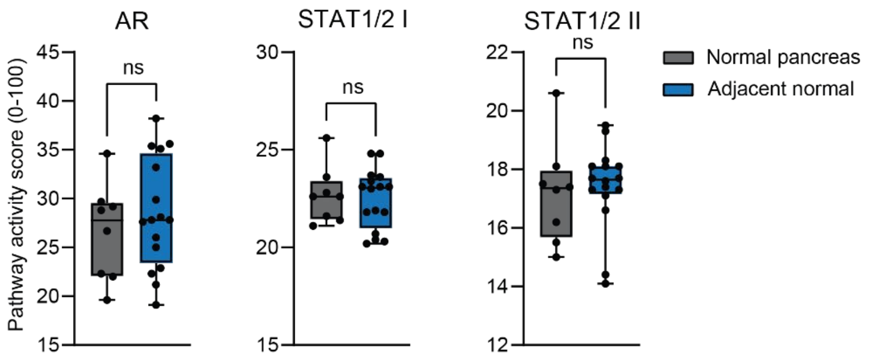

Supplemental Figure 6. Signal transduction pathway (STP) activity scores comparing normal pancreas (GSE71989) with tumor-adjacent normal samples (GSE16515). Pathways with unchanged STP activity in PDAC compared with normal tissue are depicted. A p value > 0.01 is considered non-significant (ns).

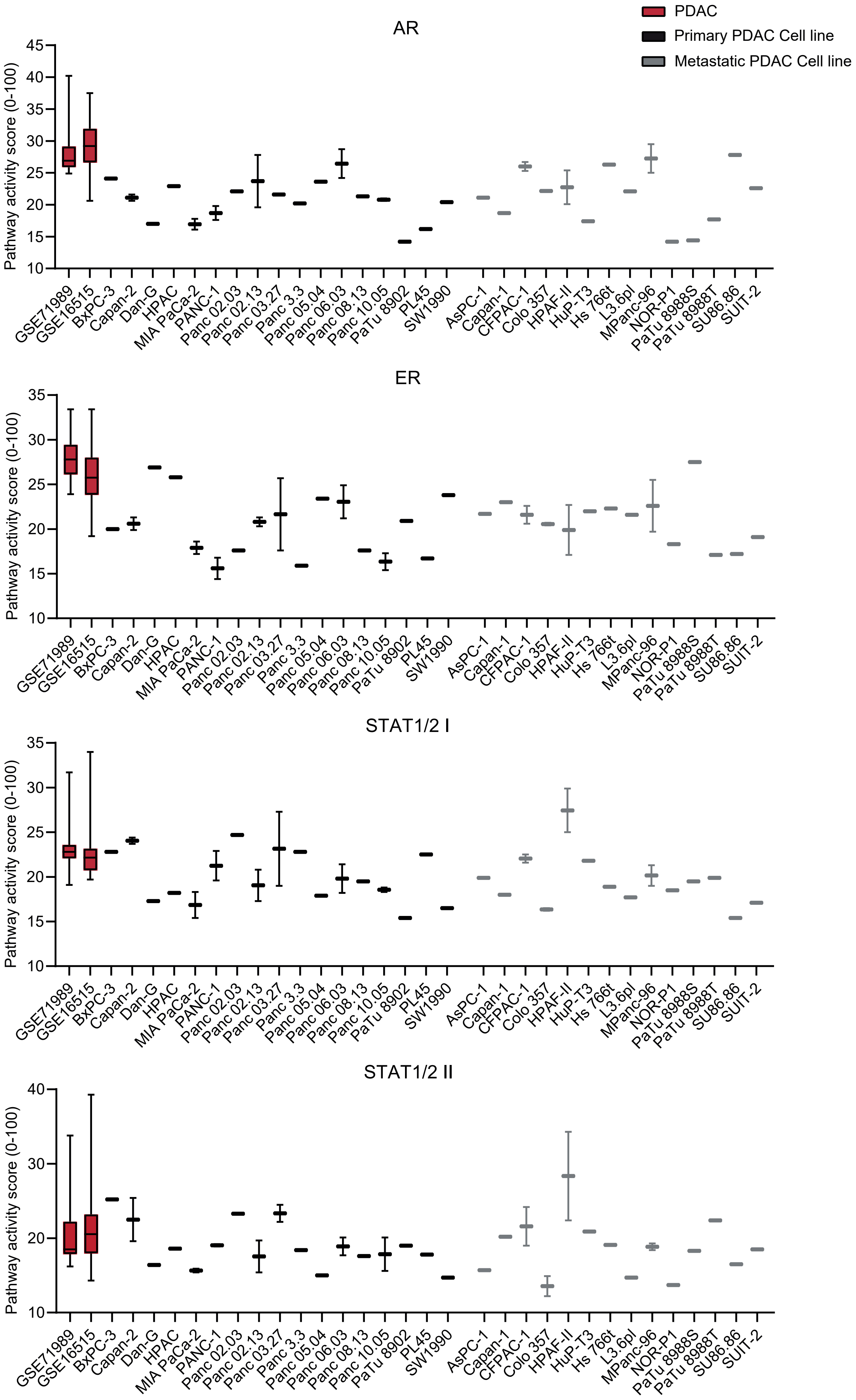

Supplemental Figure 7. Signal transduction pathway (STP) activity scores of PDAC tumor samples (GSE71989 & GSE16515) and cell lines derived from primary and metastatic PDAC.

Supplementary information

List of abbreviations

PDAC	pancreatic ductal adenocarcinoma
STP(s)	signal transduction pathway(s)
STAP	simultaneous transcriptome-based activation profiling
mRNA	messenger RNA
GEO	Gene Expression Omnibus
KRAS	Kirsten rat sarcoma virus
MAPK	mitogen-activated protein kinase
PI3K	phosphoinositide 3-kinases
FOXO	Forkhead box O
SOD2	superoxide dismutase 2
JAK-STAT	Janus kinase - signal transducer and activator of transcription
Wnt	Wingless related integration site
HH	Hedgehog
TGFβ	transforming growth factor beta
NFκB	nuclear factor kappa B
AR	androgen receptor
ER	estrogen receptor
TAAs	tumor-associated antigens
EMT	epithelial-mesenchymal transition
QC	quality control

Validation deconvolution software

Dataset	GSE54002	Validation epithelial cells	rel.cell_fibroblast_y	rel.cell_epithelial_y	rel.cell_immune_y	rel.immune_immune_y	rel.immune_t.lineage_y	rel.immune_b.lineage_y	rel.immune_granulocyte.lineage_y
**sample**	**name**	**description**	**fibroblast**	**epithelial cells**	**immune cells**	**immune cells**	**T-cell lineage**	**B-cell lineage**	**Granulocyte lineage**
**GSM1305624**	Non-tumor NMEC1	Normal mammary gland epithelium, LMD	0.01	0.98	0.01	0.14	0.17	0.14	0.54
**GSM1305625**	Non-tumor NMEC10	Normal mammary gland epithelium, LMD	0.00	0.98	0.01	0.16	0.13	0.09	0.62
**GSM1305626**	Non-tumor NMEC11	Normal mammary gland epithelium, LMD	0.01	0.97	0.02	0.16	0.19	0.11	0.54
**GSM1305627**	Non-tumor NMEC12	Normal mammary gland epithelium, LMD	0.01	0.96	0.03	0.18	0.15	0.08	0.59
**GSM1305628**	Non-tumor NMEC14	Normal mammary gland epithelium, LMD	0.01	0.98	0.02	0.14	0.15	0.10	0.61
**GSM1305629**	Non-tumor NMEC15	Normal mammary gland epithelium, LMD	0.04	0.94	0.02	0.13	0.16	0.09	0.62
**GSM1305630**	Non-tumor NMEC16	Normal mammary gland epithelium, LMD	0.01	0.97	0.02	0.08	0.10	0.03	0.79
**GSM1305631**	Non-tumor NMEC17	Normal mammary gland epithelium, LMD	0.01	0.98	0.01	0.11	0.13	0.07	0.69
**GSM1305632**	Non-tumor NMEC18	Normal mammary gland epithelium, LMD	0.00	0.98	0.02	0.12	0.18	0.05	0.65
**GSM1305633**	Non-tumor NMEC2	Normal mammary gland epithelium, LMD	0.01	0.97	0.02	0.12	0.09	0.07	0.72
**GSM1305634**	Non-tumor NMEC3	Normal mammary gland epithelium, LMD	0.01	0.96	0.03	0.23	0.24	0.10	0.43
**GSM1305635**	Non-tumor NMEC4	Normal mammary gland epithelium, LMD	0.01	0.96	0.03	0.18	0.18	0.07	0.57
**GSM1305636**	Non-tumor NMEC6	Normal mammary gland epithelium, LMD	0.00	0.96	0.04	0.22	0.20	0.09	0.49
**GSM1305637**	Non-tumor NMEC7	Normal mammary gland epithelium, LMD	0.01	0.95	0.04	0.24	0.22	0.10	0.44
**GSM1305638**	Non-tumor NMEC8	Normal mammary gland epithelium, LMD	0.01	0.98	0.01	0.11	0.12	0.10	0.68
**GSM1305639**	Non-tumor NMEC9	Normal mammary gland epithelium, LMD	0.01	0.97	0.03	0.22	0.22	0.10	0.46

Dataset	GSE164750	Validation fibroblasts and epithelial cells	rel.cell_fibroblast	rel.cell_epithelial	rel.cell_immune	rel.immune_immune	rel.immune_t.lineage	rel.immune_b.lineage	rel.immune_granulocyte.lineage
**sample**	**name**	**description**	**fibroblast**	**epithelial cells**	**immune cells**	**immune cells**	**T-cell lineage**	**B-cell lineage**	**Granulocyte lineage**
**GSM5018082**	CAF12_1	lung CAF, biological replicate 1	0.97	0.02	0.01	0.09	0.53	0.21	0.17
**GSM5018083**	CAF12_2	lung CAF, biological replicate 2	0.97	0.02	0.01	0.09	0.49	0.24	0.18
**GSM5018084**	CAF12_3	lung CAF, biological replicate 3	0.97	0.03	0.01	0.10	0.45	0.25	0.19
**GSM5018085**	CAF7_1	lung CAF, biological replicate 1	0.95	0.04	0.01	0.06	0.66	0.15	0.13
**GSM5018086**	CAF7_2	lung CAF, biological replicate 2	0.96	0.03	0.01	0.06	0.66	0.16	0.12
**GSM5018087**	CAF7_3	lung CAF, biological replicate 3	0.96	0.03	0.01	0.06	0.63	0.17	0.14
**GSM5018088**	H3122_1	non-small cell lung cancer cells	0.01	0.99	0.01	0.07	0.26	0.36	0.31
**GSM5018089**	H3122_2	non-small cell lung cancer cells	0.00	0.99	0.01	0.06	0.24	0.37	0.33
**GSM5018090**	H3122_3	non-small cell lung cancer cells	0.00	0.99	0.01	0.06	0.24	0.39	0.32
**GSM5018091**	HS5_1	normal bone marrow associated fibroblasts	0.93	0.05	0.02	0.09	0.35	0.24	0.32
**GSM5018092**	HS5_2	normal bone marrow associated fibroblasts	0.93	0.04	0.03	0.12	0.36	0.26	0.26
**GSM5018093**	HS5_3	normal bone marrow associated fibroblasts	0.93	0.04	0.02	0.13	0.40	0.27	0.21
**GSM5018094**	IMR90_1	normal lung associated fibroblasts	0.96	0.03	0.01	0.08	0.51	0.24	0.16
**GSM5018095**	IMR90_2	normal lung associated fibroblasts	0.96	0.03	0.01	0.07	0.53	0.23	0.18
**GSM5018096**	IMR90_3	normal lung associated fibroblasts	0.97	0.02	0.01	0.08	0.56	0.22	0.14
**GSM5018097**	MRC5_1	normal lung associated fibroblasts	0.93	0.05	0.01	0.07	0.56	0.21	0.15
**GSM5018098**	MRC5_2	normal lung associated fibroblasts	0.96	0.03	0.01	0.07	0.58	0.21	0.14
**GSM5018099**	MRC5_3	normal lung associated fibroblasts	0.96	0.03	0.01	0.08	0.59	0.18	0.14
**GSM5018100**	PC9_1	non-small cell lung cancer cells	0.01	0.99	0.01	0.06	0.20	0.57	0.17
**GSM5018101**	PC9_2	non-small cell lung cancer cells	0.01	0.99	0.01	0.08	0.25	0.47	0.20
**GSM5018102**	PC9_3	non-small cell lung cancer cells	0.01	0.99	0.01	0.07	0.23	0.56	0.15
**GSM5018103**	PC9_GR_1	gefitinib resistant non-small cell lung cancer cells	0.01	0.98	0.01	0.09	0.32	0.36	0.24
**GSM5018104**	PC9_GR_2	gefitinib resistant non-small cell lung cancer cells	0.02	0.98	0.01	0.10	0.32	0.36	0.22
**GSM5018105**	PC9_GR_3	gefitinib resistant non-small cell lung cancer cells	0.01	0.98	0.01	0.09	0.30	0.36	0.25
**GSM5018106**	WI38_1	normal lung associated fibroblasts	0.92	0.07	0.01	0.07	0.59	0.19	0.15
**GSM5018107**	WI38_2	normal lung associated fibroblasts	0.94	0.04	0.01	0.07	0.59	0.19	0.14
**GSM5018108**	WI38_3	normal lung associated fibroblasts	0.95	0.04	0.01	0.07	0.58	0.19	0.16

Dataset	GSE63626	Validation fibroblasts	rel.cell_fibroblast	rel.cell_epithelial	rel.cell_immune	rel.immune_immune	rel.immune_t.lineage	rel.immune_b.lineage	rel.immune_granulocyte.lineage
sample	name	description	fibroblast	epithelial cells	immune cells	immune cells	T-cell lineage	B-cell lineage	Granulocyte lineage
GSM1554131	CoSMFs3	Normal human colon submucosal fibroblasts	0.98	0.01	0.01	0.12	0.22	0.21	0.44
GSM1554132	CoSMFs4	Normal human colon submucosal fibroblasts	0.98	0.01	0.01	0.11	0.31	0.25	0.33
GSM1554133	CoSMFs8	Normal human colon submucosal fibroblasts	0.98	0.01	0.01	0.08	0.32	0.18	0.41
GSM1554134	CoSMFs11	Normal human colon submucosal fibroblasts	0.98	0.01	0.01	0.14	0.32	0.25	0.29
GSM1554135	CoSPFs3	Normal human colon subperitoneal fibroblasts	0.97	0.02	0.01	0.19	0.27	0.25	0.29
GSM1554136	CoSPFs4	Normal human colon subperitoneal fibroblasts	0.98	0.01	0.01	0.12	0.30	0.24	0.34
GSM1554137	CoSPFs8	Normal human colon subperitoneal fibroblasts	0.98	0.01	0.01	0.13	0.33	0.26	0.28
GSM1554138	CoSPFs11	Normal human colon subperitoneal fibroblasts	0.98	0.01	0.01	0.17	0.27	0.25	0.31
GSM1554139	DeFs1	Normal human breast dermal fibroblasts	0.96	0.03	0.01	0.12	0.38	0.26	0.24
GSM1554140	DeFs2	Normal human breast dermal fibroblasts	0.97	0.01	0.01	0.19	0.33	0.24	0.25
GSM1554141	DeFs3	Normal human breast dermal fibroblasts	0.98	0.01	0.01	0.11	0.36	0.28	0.24
GSM1554142	DeFs5	Normal human breast dermal fibroblasts	0.98	0.01	0.01	0.11	0.40	0.20	0.29
GSM1554143	DuSMFs3	Normal human duodenum submucosal fibroblasts	0.98	0.01	0.01	0.12	0.34	0.25	0.29
GSM1554144	DuSMFs4	Normal human duodenum submucosal fibroblasts	0.99	0.01	0.01	0.09	0.33	0.24	0.33
GSM1554145	DuSMFs5	Normal human duodenum submucosal fibroblasts	0.98	0.01	0.01	0.10	0.28	0.26	0.36
GSM1554146	DuSPFs3	Normal human duodenum subperitoneal fibroblasts	0.99	0.01	0.01	0.09	0.30	0.24	0.37
GSM1554147	DuSPFs4	Normal human duodenum subperitoneal fibroblasts	0.99	0.01	0.01	0.10	0.35	0.25	0.30
GSM1554148	DuSPFs5	Normal human duodenum subperitoneal fibroblasts	0.99	0.01	0.01	0.11	0.31	0.25	0.34
GSM1554149	EsSMFs1	Normal human esophagus submucosal fibroblasts	0.98	0.01	0.01	0.13	0.33	0.26	0.28
GSM1554150	EsSMFs4	Normal human esophagus submucosal fibroblasts	0.99	0.01	0.01	0.14	0.33	0.31	0.22
GSM1554151	EsSMFs7	Normal human esophagus submucosal fibroblasts	0.99	0.01	0.01	0.15	0.32	0.27	0.26
GSM1554152	EsSPFs1	Normal human esophagus subperitoneal fibroblasts	0.95	0.05	0.01	0.10	0.36	0.23	0.31
GSM1554153	EsSPFs4	Normal human esophagus subperitoneal fibroblasts	0.98	0.01	0.01	0.15	0.36	0.28	0.21
GSM1554154	EsSPFs7	Normal human esophagus subperitoneal fibroblasts	0.97	0.02	0.01	0.12	0.33	0.29	0.26
GSM1554155	GaFs1	Normal human gallbladder fibroblasts	0.98	0.01	0.01	0.08	0.29	0.17	0.46
GSM1554156	GaFs2	Normal human gallbladder fibroblasts	0.98	0.01	0.01	0.04	0.21	0.10	0.65
GSM1554157	GaFs4	Normal human gallbladder fibroblasts	0.98	0.01	0.01	0.06	0.32	0.19	0.43
GSM1554158	HSCs1	Normal human hepatic stellate cells	0.98	0.01	0.01	0.06	0.33	0.18	0.42
GSM1554159	HSCs2	Normal human hepatic stellate cells	0.96	0.03	0.01	0.07	0.16	0.11	0.65
GSM1554160	ILSMFs1	Normal human ileum submucosal fibroblasts	0.98	0.01	0.01	0.12	0.31	0.26	0.31
GSM1554161	ILSMFs3	Normal human ileum submucosal fibroblasts	0.97	0.02	0.01	0.11	0.33	0.25	0.31
GSM1554162	ILSMFs5	Normal human ileum submucosal fibroblasts	0.97	0.02	0.01	0.13	0.31	0.27	0.29
GSM1554163	ILSPFs1	Normal human ileum subperitoneal fibroblasts	0.98	0.01	0.01	0.12	0.32	0.29	0.27
GSM1554164	ILSPFs3	Normal human ileum subperitoneal fibroblasts	0.98	0.01	0.01	0.15	0.35	0.27	0.23
GSM1554165	ILSPFs5	Normal human ileum subperitoneal fibroblasts	0.98	0.01	0.01	0.13	0.32	0.27	0.27
GSM1554166	LiFs2	Normal human liver fibroblasts	0.98	0.01	0.01	0.10	0.23	0.21	0.45
GSM1554167	LiFs6	Normal human liver fibroblasts	0.98	0.02	0.01	0.10	0.39	0.24	0.28
GSM1554168	LiFs7	Normal human liver fibroblasts	0.98	0.01	0.01	0.11	0.28	0.24	0.37
GSM1554169	LuFs3	Normal human lung fibroblasts	0.97	0.02	0.01	0.10	0.48	0.22	0.21
GSM1554170	LuFs4	Normal human lung fibroblasts	0.98	0.02	0.01	0.10	0.46	0.22	0.22
GSM1554171	LuFs5	Normal human lung fibroblasts	0.97	0.02	0.01	0.12	0.41	0.24	0.23
GSM1554172	MaFs1	Normal human mammary fibroblasts	0.95	0.04	0.01	0.13	0.41	0.24	0.21
GSM1554173	MaFs2	Normal human mammary fibroblasts	0.97	0.02	0.01	0.14	0.38	0.28	0.20
GSM1554174	MaFs3	Normal human mammary fibroblasts	0.97	0.02	0.01	0.12	0.41	0.27	0.20
GSM1554175	MaGFs1	Normal human mammary gland fibroblasts	0.94	0.05	0.01	0.15	0.32	0.28	0.26
GSM1554176	MaGFs3	Normal human mammary gland fibroblasts	0.92	0.07	0.01	0.10	0.47	0.22	0.21
GSM1554177	MaGFs4	Normal human mammary gland fibroblasts	0.93	0.07	0.01	0.10	0.50	0.22	0.19
GSM1554178	MaGFs5	Normal human mammary gland fibroblasts	0.88	0.11	0.01	0.12	0.39	0.27	0.21
GSM1554179	PrFs1	Normal human prostate fibroblasts	0.96	0.03	0.01	0.12	0.44	0.25	0.19
GSM1554180	PrFs2	Normal human prostate fibroblasts	0.98	0.01	0.01	0.12	0.41	0.26	0.21
GSM1554181	PrFs3	Normal human prostate fibroblasts	0.97	0.02	0.01	0.13	0.39	0.27	0.22
GSM1554182	StSMFs2	Normal human stomach submucosal fibroblasts	0.98	0.01	0.01	0.09	0.28	0.18	0.45
GSM1554183	StSMFs9	Normal human stomach submucosal fibroblasts	0.97	0.02	0.01	0.17	0.30	0.30	0.23
GSM1554184	StSMFs10	Normal human stomach submucosal fibroblasts	0.98	0.01	0.01	0.18	0.28	0.28	0.26
GSM1554185	StSPFs2	Normal human stomach subperitoneal fibroblasts	0.98	0.01	0.01	0.11	0.42	0.24	0.24
GSM1554186	StSPFs9	Normal human stomach subperitoneal fibroblasts	0.98	0.01	0.01	0.12	0.39	0.24	0.25
GSM1554187	StSPFs11	Normal human stomach subperitoneal fibroblasts	0.98	0.01	0.01	0.14	0.31	0.28	0.26
GSM1554188	UtFs1	Normal human uterine fibroblasts	0.95	0.04	0.01	0.19	0.33	0.28	0.20
GSM1554189	UtFs2	Normal human uterine fibroblasts	0.94	0.05	0.01	0.17	0.35	0.28	0.20
GSM1554190	UtFs3	Normal human uterine fibroblasts	0.92	0.07	0.01	0.19	0.34	0.27	0.20
GSM1554191	VAFs2	Normal human vascular adventitial fibroblasts	0.98	0.02	0.01	0.12	0.31	0.29	0.28
GSM1554192	VAFs3	Normal human vascular adventitial fibroblasts	0.97	0.02	0.00	0.10	0.28	0.31	0.30
GSM1554193	VAFs4	Normal human vascular adventitial fibroblasts	0.98	0.01	0.01	0.12	0.33	0.32	0.24

Dataset	GSE28490	Validation immune cells and subsets	rel.cell_fibroblast_x	rel.cell_epithelial_x	rel.cell_immune_x	rel.immune_immune_x	rel.immune_t.lineage_x	rel.immune_b.lineage_x	rel.immune_granulocyte.lineage_x
**sample**	**name**	**description**	**fibroblast**	**epithelial cells**	**immune cells**	**immune cells**	**T-cell lineage**	**B-cell lineage**	**Granulocyte lineage**
GSM705287	Monocytes rep1	CD14+ monocytes	0.00	0.01	0.99	0.40	0.01	0.01	0.58
GSM705288	Monocytes rep2	CD14+ monocytes	0.00	0.01	0.99	0.37	0.03	0.05	0.56
GSM705289	Monocytes rep3	CD14+ monocytes	0.00	0.01	0.99	0.41	0.03	0.02	0.54
GSM705290	Monocytes rep4	CD14+ monocytes	0.00	0.01	0.99	0.42	0.04	0.03	0.52
GSM705291	Monocytes rep5	CD14+ monocytes	0.00	0.01	0.99	0.42	0.01	0.02	0.55
GSM705292	Monocytes rep6	CD14+ monocytes	0.00	0.01	0.99	0.41	0.02	0.02	0.55
GSM705293	Monocytes rep7	CD14+ monocytes	0.00	0.01	0.99	0.42	0.02	0.01	0.55
GSM705294	Monocytes rep8	CD14+ monocytes	0.00	0.01	0.99	0.41	0.01	0.03	0.55
GSM705295	Monocytes rep9	CD14+ monocytes	0.00	0.01	0.99	0.40	0.01	0.01	0.58
GSM705296	Monocytes rep10	CD14+ monocytes	0.00	0.01	0.99	0.41	0.01	0.02	0.56
GSM705297	B cells rep1	CD19+ B cells	0.00	0.01	0.99	0.31	0.01	0.68	0.00
GSM705298	B cells rep2	CD19+ B cells	0.00	0.01	0.98	0.34	0.01	0.64	0.01
GSM705299	B cells rep3	CD19+ B cells	0.00	0.01	0.99	0.31	0.01	0.68	0.00
GSM705300	B cells rep4	CD19+ B cells	0.00	0.01	0.99	0.33	0.01	0.66	0.01
GSM705301	B cells rep5	CD19+ B cells	0.00	0.01	0.99	0.32	0.00	0.67	0.00
GSM705302	CD4+ T cells rep1	CD4+ T cells	0.00	0.01	0.99	0.47	0.47	0.03	0.03
GSM705303	CD4+ T cells rep2	CD4+ T cells	0.00	0.01	0.99	0.40	0.36	0.02	0.22
GSM705304	CD4+ T cells rep3	CD4+ T cells	0.00	0.01	0.99	0.46	0.41	0.02	0.12
GSM705305	CD4+ T cells rep4	CD4+ T cells	0.00	0.01	0.99	0.46	0.48	0.03	0.02
GSM705306	CD4+ T cells rep5	CD4+ T cells	0.00	0.01	0.99	0.48	0.47	0.03	0.02
GSM705312	CD8+ T cells rep1	CD8+ T cells	0.00	0.01	0.99	0.48	0.50	0.01	0.01
GSM705313	CD8+ T cells rep2	CD8+ T cells	0.00	0.01	0.99	0.45	0.53	0.01	0.01
GSM705314	CD8+ T cells rep3	CD8+ T cells	0.00	0.01	0.99	0.44	0.55	0.01	0.01
GSM705315	CD8+ T cells rep4	CD8+ T cells	0.00	0.01	0.99	0.48	0.50	0.01	0.01
GSM705316	CD8+ T cells rep5	CD8+ T cells	0.00	0.01	0.99	0.48	0.50	0.01	0.01
GSM705307	NK cells rep1	CD56+ NK cells	0.00	0.01	0.99	0.87	0.08	0.03	0.02
GSM705308	NK cells rep2	CD56+ NK cells	0.00	0.01	0.99	0.90	0.06	0.03	0.01
GSM705309	NK cells rep3	CD56+ NK cells	0.00	0.01	0.99	0.91	0.05	0.03	0.02
GSM705310	NK cells rep4	CD56+ NK cells	0.00	0.01	0.99	0.90	0.06	0.03	0.01
GSM705311	NK cells rep5	CD56+ NK cells	0.00	0.01	0.99	0.90	0.06	0.04	0.01
GSM705326	Neutrophils rep1	Human Neutrophil Enrichment Kit	0.00	0.01	0.99	0.45	0.01	0.01	0.53
GSM705327	Neutrophils rep2	Human Neutrophil Enrichment Kit	0.00	0.01	0.99	0.45	0.01	0.01	0.53
GSM705328	Neutrophils rep3	Human Neutrophil Enrichment Kit	0.00	0.01	0.99	0.51	0.01	0.01	0.47
GSM705317	Eosinophils rep1	Human Eosinophil Enrichment Kit	0.00	0.01	0.99	0.93	0.02	0.04	0.02
GSM705318	Eosinophils rep2	Human Eosinophil Enrichment Kit	0.00	0.01	0.99	0.93	0.02	0.03	0.02
GSM705319	Eosinophils rep3	Human Eosinophil Enrichment Kit	0.00	0.01	0.99	0.94	0.01	0.03	0.01
GSM705320	Eosinophils rep4	Human Eosinophil Enrichment Kit	0.00	0.01	0.99	0.94	0.02	0.03	0.01
GSM705321	mDC rep1	CD11c+ myeloid dendritic cells (mDCs)	0.00	0.02	0.98	0.87	0.04	0.04	0.05
GSM705322	mDC rep2	CD11c+ myeloid dendritic cells (mDCs)	0.00	0.02	0.98	0.86	0.05	0.03	0.06
GSM705323	mDC rep3	CD11c+ myeloid dendritic cells (mDCs)	0.00	0.02	0.98	0.87	0.06	0.04	0.04
GSM705324	mDC rep4	CD11c+ myeloid dendritic cells (mDCs)	0.00	0.02	0.98	0.85	0.06	0.03	0.06
GSM705325	mDC rep5	CD11c+ myeloid dendritic cells (mDCs)	0.00	0.02	0.98	0.83	0.06	0.04	0.06
GSM705329	pDC rep1	CD123+ plasmacytoid dendritic cells (pDCs)	0.00	0.15	0.85	0.81	0.05	0.09	0.06
GSM705330	pDC rep2	CD123+ plasmacytoid dendritic cells (pDCs)	0.00	0.07	0.92	0.84	0.03	0.11	0.03
GSM705331	pDC rep3	CD123+ plasmacytoid dendritic cells (pDCs)	0.00	0.12	0.87	0.85	0.03	0.09	0.03
GSM705332	pDC rep4	CD123+ plasmacytoid dendritic cells (pDCs)	0.00	0.08	0.92	0.87	0.03	0.08	0.03
GSM705333	pDC rep5	CD123+ plasmacytoid dendritic cells (pDCs)	0.00	0.08	0.92	0.85	0.03	0.09	0.03

Validation of deconvolution software on pure cell populations from 4 GEO mRNA datasets. Relative cell content in the samples is calculated based on lineage specific mRNA expression with 1.0 as 100%. From the immune cell content, relative percentages of immune cell subsets are calculated.

Overview of STP analysis results from the Affymetrix datasets described in the source publications

1. GSE15471

Samples: 36 pairs of PDAC and normal adjacent pancreatic tissue. The samples were obtained directly after surgery and snap-frozen in liquid nitrogen. Diagnosis was performed by an experienced pathologist. Gene expression profiling was performed. Five adjacent normal samples showed a gene expression closer related to the tumor samples. Histopathological analyses revealed either tumoral infiltration and/or fibrotic reaction in these normal samples, which makes it difficult to differentiate them from tumor tissue.

Data analysis: differential gene expression.

No pathway activity analysis. Some genes involved in signaling transduction pathways were measured.

The study tested a set of 400 genes and except for three genes, all were upregulated. From these genes, 65 had p-values lower than 10^-14 and at least 2-fold overexpression in the tumors when compared to the normal tissues. Overexpression of the genes ANXA2, ANXA2P2, KRT7, MAP4K4, LAMC2, OACT2, SLC16A3, TM4SF1 and KYNU was inversely correlated with patient survival. Genes involved in epithelial-mesenchymal transition were overexpressed: LAMC2, HOXB7, MAP4K4, FN1, PLAU, POSTN AND LEF1.

2. GSE16515

Samples: 19 pairs of PDAC and adjacent normal pancreatic tissue. Samples were obtained after surgical resection, timing and method of preservation is not specified. Three samples were excluded due to poor quality.

Data analysis: differential gene expression.

Gene expression profiling was performed, expression levels were normalized by GCRMA

Pathway activity analysis: No pathway activity analysis. Ingenuity pathway analysis was performed on Akt and parts of pathways involved in the PI3K and MAPK pathways.

Gene signatures between normal tissue and PDAC were compared. Network analysis with ingenuity pathway analysis on the most differentiate expressed genes between normal tissue and PDAC showed that the network around Akt was the top network in PDAC. FKPB51 gene expression levels

were lower in PDAC when compared to normal tissue. FKPB51 might act as tumor suppressor, as samples with lower or loss of FKBP51 showed higher Akt/GSK-3β phosphorylation.

Experiments with PDAC cell lines identified FKBP51 as an important determinant of cancer cell response to clinically important chemotherapeutic agents in cell lines. Decreased expression of FKBP51 caused resistance to chemotherapy in cancer cell lines. FKBP51 acts as a scaffolding protein for Akt and PHLPP, thereby enhancing the phosphatase activity of PHLPP toward Akt.

Overall the results suggest FKPB51 negatively regulates Akt expression and loss of FKPB51 causes hyperactivation of Akt resulting in disease progression and resistance to chemotherapy.

3. GSE32676

Samples: 25 PDAC tumor samples from early stage PDAC and 7 non-malignant samples snap-frozen after surgery. Characteristics and origin of 7 non-malignant pancreas samples is not specified. Non-malignant pancreas samples could have been derived from adjacent normal pancreas from PDAC patients.

Data analysis: differential gene expression.

Pathway analysis: No pathway activity analysis. Genes in the PI3K/Akt and SRC signaling pathways were measured. Gene expression signature was determined from PDAC compared to benign pancreas samples. Survival based pathway and gene ontology analysis was performed on this gene signature. Several survival-correlated genes linked with poor prognosis upregulated the PI3K/Akt and SRC signaling pathways. For example, high expression of EGFR was linked with poor prognosis, which activates the PI3K pathway. In conclusion: EGFR, SRC signaling, and PI3K/AKT pathway activation are strongly linked to clinical disease progression. Two discrete subsets of pancreatic tumors characterized by either SRC or PI3K/AKT signaling that may dictate variable responses to targeted therapy.

4. GSE71989

Samples: 8 normal pancreas tissues from tissue donors and 14 PDAC tissues obtained after surgery. Timing after surgery and method of isolation are not specified.

Normal pancreas samples were from donors who did not have pancreatic disease. Causes of death were sudden death (2x), heart disease, cerebral vascular accident, head trauma, emphysema, failure to thrive and was unknown in one case.

Data analysis: differential gene expression.

No signal transduction pathway activity analysis.

Gene expression profiling was performed to determine the alterations/upregulation of transcribed ultraconserved regions (T-UCRs) in PDAC. 175 out of 307 expressed T-UCRs in pancreas tissues were differently expressed in PDAC when compared to benign pancreas. All 175 of the differentially expressed T-UCRs were increased in the PDAC and none were decreased.

Culture conditions cell line datasets

1. GSE17891

All cell lines were cultured in DMEM with 10% FBS in 5% CO2 on plastic

2. GSE21654

22 pancreatic cancer cell lines were grown to 80% confluence in DMEM/FBS/PenStrep media and then harvested for total RNA
